# Hansen’s Disease in Ecuador: Current Status, Knowledge Gaps, and Research Priorities: A Literature Review

**DOI:** 10.3390/pathogens14080832

**Published:** 2025-08-21

**Authors:** Manuel Calvopiña, Juan S. Izquierdo-Condoy, Esteban Ortiz-Prado, Jorge Vasconez-Gonzalez, Lorena Vaca, Elías Guamán

**Affiliations:** 1 One Health Research Group, Facultad de Medicina, Universidad De Las Américas (UDLA), Quito 170124, Ecuador; juan.izquierdo.condoy@udla.edu.ec (J.S.I.-C.); esteban.ortiz.prado@udla.edu.ec (E.O.-P.); jorge.vasconez.gonzalez@udla.edu.ec (J.V.-G.); 2 Servicio de Dermatología, Hospital General Docente de Calderon, Ministerio de Salud Pública, Quito 170201, Ecuador; wilorenvp29@hotmail.com; 3 Hospital General “Marco Vinicio Iza”, Ministerio de Salud Pública, Nueva Loja 170702, Ecuador; eliassguaman@gmail.com

**Keywords:** leprosy, Hansen’s disease, epidemiology, literature review, current status, update, Ecuador

## Abstract

**Background:** Hansen’s disease or leprosy is one of the 21 neglected tropical diseases (NTDs). In Ecuador, leprosy is considered eliminated as a public health problem; however, new cases are reported annually. Additionally, *Mycobacterium leprae* infection was detected in nine-banded armadillos across the country, suggesting a potential zoonotic reservoir. This literature review aims to provide an updated overview of the epidemiological situation of leprosy in Ecuador, identify knowledge gaps, and outline research priorities to support the development of a comprehensive national strategy for achieving zero autochthonous cases. **Methods:** This article analyses the current situation of leprosy in Ecuador based on international and national publications. A retrospective literature search using five international, regional, and national publications on leprosy published between 1954 and 2024 (70 years) with no restriction on language or publication date, was performed. **Findings:** Our review identified 28 publications with the earliest article dating back to 1954. Of these, 14 were published in international journals, 15 (53.6%) were in Spanish. Four nationwide studies documented leprosy cases across Ecuador’s three continental regions (Coast, Andes, and Amazon) with a predominance in the tropical coast. No cases have been reported from the Galápagos Islands. From 1983, Ecuador started multi-drug therapy. Data from the Ministry of Public Health (MoH) system identified 1539 incident cases, showing a significant decline in new cases from 2000 to 2024, with no cases in children. New cases detection rate by 100,000 inhabitants was 0.51 in 2019 according to the World Health Organization (WHO). No study has genotyped the *Mycobacterium* spp. in human cases, other animal species, or environment. According to the MoH, multibacillary leprosy accounts for 78.95% of diagnosed cases, with confirmation based on Ziehl–Neelsen staining and histopathology. No survey has assessed disabilities, knowledge, attitudes, and practices (KAP) or stigma related to leprosy. Research is needed on transmission routes, *Mycobacterium* genotyping, genetic susceptibility, and antibiotic resistance. BCG vaccination coverage fell to 75.3% in 2021. Cases are currently diagnosed and treated on an outpatient basis in large hospitals. **Conclusions:** This comprehensive review highlights persistent gaps in leprosy research and critical information, despite seven decades of documented cases in Ecuador. The disease is still endemic across the country, particularly at subnational level in the subtropics and tropics of the Pacific coast and the Amazon. There is a need for nationwide epidemiological research on reservoirs and the environment applying the One Health concept. Increased laboratory facilities and readily available official data are required to improve our understanding of leprosy in Ecuador. Strengthening community-level efforts is essential for Ecuador to meet the targets of the “WHO’s Towards Zero Leprosy: Strategy 2021–2030.”

## 1. Introduction

Hansen’s disease or leprosy is 1 of the 21 neglected tropical diseases (NTDs), a group of conditions prevalent in tropical regions. Leprosy has been known to mankind since ancient times. In the “WHO Roadmap for NTDs 2021–2030”, leprosy, human African trypanosomiasis, and onchocerciasis are targeted for interruption of transmission [1]. Ecuador achieved the elimination of leprosy as a public health problem, defined as less than 1 case per 10,000 people; this was achieved globally in 2000. Recently, the WHO “Global Leprosy Strategy 2021–2030” has focused the new strategy on “interrupting transmission and achieving zero autochthonous cases, zero disability, zero stigma and discrimination” [2]. In doing so, the strategy aims to motivate high-burden countries to accelerate activities while compelling low-burden countries to complete the unfinished task of making leprosy history. However, pockets of endemicity have continued in many countries, particularly in rural remote jurisdictions, like in Ecuador [3,4]. The verification process for interruption of transmission and elimination of leprosy begins at the subnational level. Hence, updated published information on leprosy epidemiology for each country is needed. New tools such as the leprosy elimination monitoring tool and the leprosy program and transmission assessment tool have been introduced by the WHO to monitor epidemiological situations for every country [5]. Therefore, high-quality data collection and reporting are essential to monitor progress towards those milestones.

Internationally Ecuador is considered endemic for leprosy [5], confirmed by the Ecuadorian Ministry of Public Health (MoH) and by our latest publication [4,6]. The number of cases has substantially decreased in the last decade, cases are concentrated in older adults and not in children, prevailing in the tropics and subtropics of continental landmass. In Ecuador, leprosy is of immediate notification, through the Individual EPI 1 form, using the instrument of the Integral System of ViEpi surveillance [6]. Recent new cases of leprosy are reported in the literature and by the MoH [6,7,8,9,10,11,12].

To further understand the epidemiological situation and other relevant contextual factors, identify knowledge gaps, and outline research priorities of leprosy in Ecuador, we conducted this bibliographic study, addressing all available evidence from publications in national and international journals. Theses, official publications, and clinical cases were also included in our revision.

## 2. Geographical Location and Populations of Ecuador

**Figure 1:**
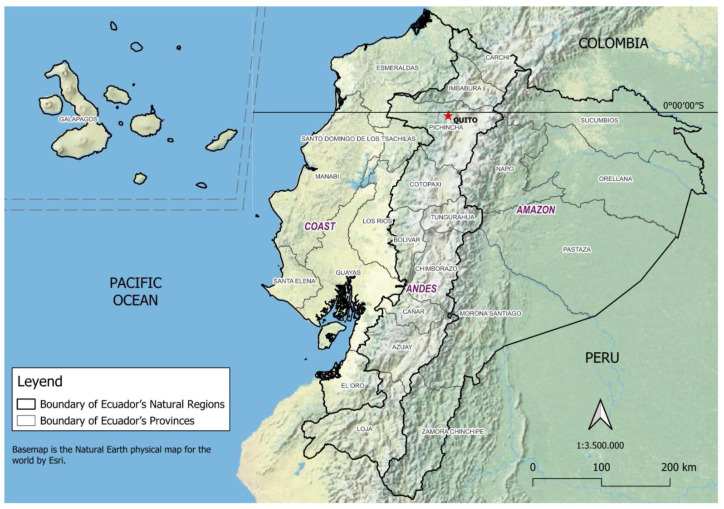
Geographic and administrative map of Ecuador. Ecuador is bordered by Colombia to the north, Peru to the south and east, and the Pacific Ocean to the west. Comprises 4 main geographical regions: the temperate Andes (center), the Pacific coastal region (west), the Amazon (east), and the Galápagos Islands, located approximately 1000 km off the mainland in the Pacific Ocean. Ecuador is administratively divided into 24 provinces and 224 cantons, with cantons functioning as second-level subdivisions. The coast encompasses 7 provinces (Esmeraldas, Manabí, Santa Elena, Guayas, Santo Domingo de los Tsáchilas, Los Ríos, and El Oro); the Andes includes 10 provinces (Azuay, Bolívar, Cañar, Carchi, Chimborazo, Cotopaxi, Imbabura, Loja, Pichincha, and Tungurahua); and the Amazon comprises 6 provinces (Sucumbíos, Orellana, Napo, Pastaza, Morona Santiago, and Zamora Chinchipe).

Ecuador is in the northwestern of South America and straddles both the Equator and the Andes Mountains. The Andes cross the country from north to south and divide it into 3 different natural regions: the Pacific coast with subtropical and tropical lowlands, the Andean temperate region with highlands and valleys and, in the east, the Amazon lowlands covered by humid tropical rain forest; in addition, there is the Galápagos Islands (Figure 1). Ecologically, Ecuador is an extremely diverse country with a total area of 283,560 km^2^, ranging from tropical to nival (permanent snow) and from the rainforest to desert brush. Ecuador is considered as a medium-income economy nation [13], with 64% of the land considered to be subtropical and tropical climate. According to the latest census data, the total population of Ecuador is 16,938,986 people. The coastal region concentrates 53.3% of the population, the Andean 41.04%, the Amazon 5.48%, and the Galápagos Islands 0.17%. A total of 63.1% of the population is urban. Ecuador is a multiethnic nation with a diverse population. The main ethnic groups include Mestizo, Indigenous, Afro-Ecuadorian, Montubio, and White, with Mestizos forming the largest group. Indigenous peoples are comprised of 14 nationalities, each with distinct cultures and languages [14].

## 3. Historical Background

The first known written publication of Hansen’s disease in Ecuador dates back to 1954 [15]. According to a 1967 publication, several sanatoriums or leprosarium—locally known as *leprocomios*—were operating in Ecuador. These included “Verde Cruz” in Quito, with a capacity of 120 beds, the “Mariano Estrella” in Cuenca with 60 beds, and the Buza dispensary in El Oro Province. Additionally, in Guayaquil, a 12-bed transit facility for leprosy patients was converted in 1956 into the “Dermatological Dispensary” [16]. Patients in these *leprocomios* were isolated from their families and society, often without access to adequate wound care, treatment, or rehabilitation services.

Historically, a Spanish Royal Decree from 1679 required the Royal Audience of Quito and the provinces of Guayaquil and Cuenca to establish isolation facilities for people with leprosy. As a result, the “*Leprocomio* de la Misericordia” was founded in 1816 in Perezpata, Cuenca. Due to the increasing number of cases, the “Hacienda del Jordán” was converted into a leprosarium in 1835, followed by the establishment of a women’s facility in Miraflores in 1854. In 1889, the *leprocomios* came under the administration of Dominican nuns from France, who arrived to fulfil what was described as “a noble and humane mission.”

According to a 1956 report, Ecuador had established a National Leprosy Service, which reported cases to the General Directorate of Health. Officials Badger L.F., Hernández D., and Rendón L. documented 372 cases registered from 1948. By 1956, an additional 60 cases had been reported, bringing the total to 492 [17]. A 1962 publication noted that 250 patients were undergoing treatment across the four *leprocomios*. Among these, 71 (9.1%) were children under 15 years of age [16]. Most cases were from the coastal provinces of Guayas, Los Ríos, El Oro, Azuay, and Loja [15,16,17].

In response to the growing burden, an active leprosy control program was launched in 1962 by the General Directorate of Health with support from Pan American Health Organization (PAHO) and United Nations Children’s Fund (UNICEF) [18]. However, this initiative was discontinued after two years. Subsequently, the “National Leprosy Control Program” was formally established [19]. In 1957, several recommendations were proposed for leprosy control: (1) recognizing leprosy as a tropical public health disease and allocating budget for its management under Ecuadorian National Health Legislation; (2) classifying it as a notifiable disease and including it among major endemics such as malaria, plague, yellow fever, and rabies; (3) enforcing the isolation of affected individuals; (4) separating children from leprosy patients; (5) preventing the entry of infected individuals from foreign countries; (6) requiring BCG vaccination for children; (7) establishing centers to receive newborns of mothers with leprosy; (8) launching public education campaigns to reduce stigma; and (9) providing training on leprosy for students and healthcare personnel [20]. These measures were updated by the Ministry of Health in 2014 in accordance with PAHO recommendations [21].

Historically, leprosy was classified as “malignant” (lepromatous leprosy) or “benign” (tuberculoid leprosy) [15,16,17]. Lepromin antigen was locally developed from lymphoid nodule tissue obtained from an Ecuadorian patient [15].

Diagnostic methods used up to 1967 included the Mitsuda (lepromin) skin test, bacilloscopy using Ziehl–Neelsen staining, and histopathology of skin biopsies [15,17]. During the two-year active case detection campaign led by the MoH, PAHO, and UNICEF, 4293 contacts were registered, 2772 examined, and 2027 bacilloscopies performed [16]. Two studies employed serological detection of anti-PGL-1 and LAM-B antibodies [22,23]. Subsequently, and up to the present, the recommendations of the WHO/PAHO, and MoH are followed [21,24]. To date, one original research has used qPCR to detect *M. leprae* and *M. lepromatosis* in armadillos [25].

Treatment was initially limited to 100 mg tablets of dapsone (DDS). Since 1983, Ecuador has adopted WHO/PAHO-recommended multidrug therapy (MDT), including rifampicin, clofazimine, and dapsone. With support from PAHO and the German Aid for Leprosy Patients (AYU), the country achieved 100% MDT coverage by 1987 [19].

In 1984, the MoH published a Manual of Standards for the Control of Hansen’s Disease (Manual de Normas para Control de la Enfermedad de Hansen, in Spanish), establishing national guidelines for diagnosis, treatment, and control. Ecuador employed both the Madrid and Ridley–Jopling classifications [19]. Later, the International Classification of Diseases (ICD-10) was adopted. Currently, the WHO classification of multibacillary (MB) and paucibacillary (PB) leprosy is used [26].

Since 2014, leprosy has been designated as an immediately notifiable disease in Ecuador, reported weekly using the ViEpi surveillance system via the Individual EPI 1 form [6]. That same year, the MoH released the Manual of Procedures of the Epidemiological Surveillance Subsystem (SIVE-ALERTA), which defines case criteria, diagnostic procedures, and control measures [21]. Hospitalized cases are recorded by the National Institute of Statistics and Censuses (INEC) and are publicly available online [https://www.ecuadorencifras.gob.ec/camas-y-egresos-hospitalarios/ (accessed on 14 August 2025)].

All diagnosed cases are required to receive outpatient treatment following PAHO guidelines [24], with hospitalization limited to severe reactional states. Currently, suspected cases are examined by specialists in dermatology departments of public hospitals, where diagnosis and treatment are provided free of charge. Blister-pack formulations for PB and MB cases are distributed free of charge, but only in specialized or provincial hospitals with dermatological services. The MoH manages MDT procurement and distribution nationally. Diagnosed individuals must attend local health centers monthly to collect their medications [21].

**Figure 2:**
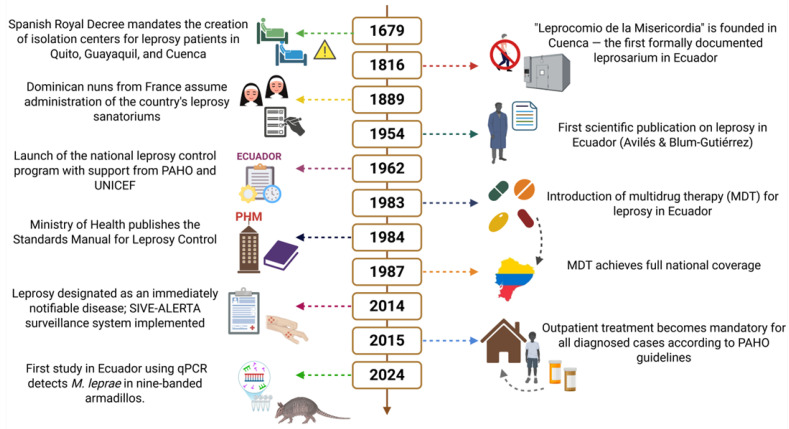
Timeline of key milestones in the history of Hansen’s disease in Ecuador (1679–2024).

To better contextualize the historical trajectory of leprosy in Ecuador, Figure 2 presents a timeline highlighting key milestones from colonial-era decrees to modern surveillance and treatment strategies. This visual synthesis illustrates the evolution of institutional responses, diagnostic practices, and therapeutic interventions over nearly two centuries, underscoring both the persistence of the disease and the shifting paradigms in its management.

## 4. Study Design, Methods, and Ethics

This study is a comprehensive narrative literature review with exploratory and descriptive components. We conducted a structured and exhaustive literature search without language or time restrictions using the following academic databases: PubMed, Web of Science, Scopus, SciELO, LILACS, and LATINDEX. Additionally, we searched Google Scholar for complementary sources and manually screened references cited in key publications. Non-indexed local journals, bulletins, theses, official publications, and clinical cases were included in our analysis. We reviewed the published literature with anonymized data and, thus, the study does not require any ethical approval. There were no patients and populations involved in this review.

## 5. Results

### 5.1. The Published Literature in Ecuador

**Figure 3:**
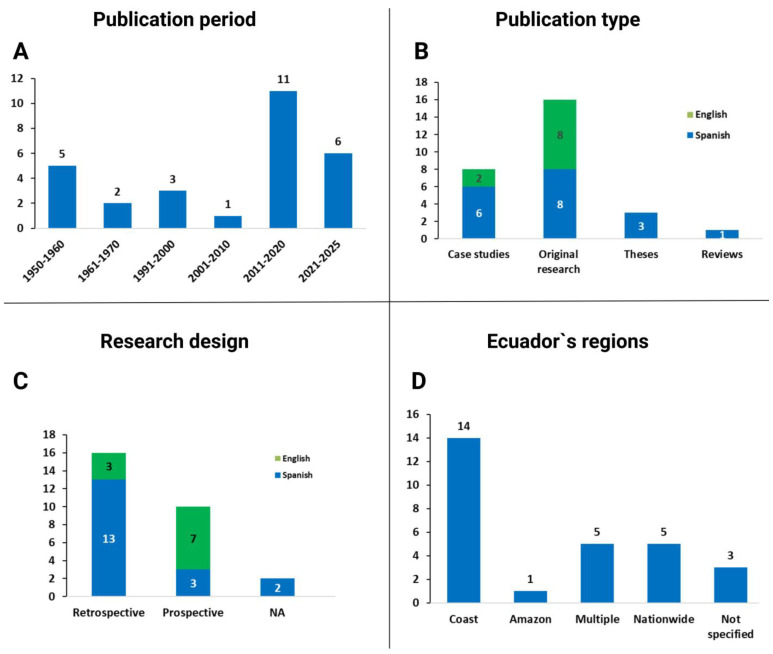
Statistics of the 28 publications on leprosy in Ecuador (1954–2024). (**A**) shows the distribution of publications by decade; (**B**) displays the types of publications, indicating language—English (green bars) and Spanish (blue bars); (**C**) summarizes the study designs; and (**D**) categorizes the studies by ecoregion. “Multiple” refers to studies conducted in more than one ecoregion simultaneously, while “Nationwide” indicates studies covering the entire country. “Case studies” include both case reports and case series. For additional details see Supplemental Table S1.

A total of 28 publications were identified between 1954 and 2024. Of these, 39.3% (n = 11) were published during the period 2011–2020, while no publications were found between 1971 and 1990. Sixteen were original research (eight in English, eight in Spanish), eight were case reports or case series (six in Spanish and two in English), three were theses (all in Spanish), and one was a narrative review in Spanish. Regarding study design, 16 were retrospective, 10 prospective, and 2 did not specify the design. Fourteen studies were published in international journals, of which ten were in English. Geographically, half (n = 14) were conducted on the coast (Figure 3). Most studies (n = 6, 92.9%) focused on human cases, while one focused on armadillos.

### 5.2. Geographic Distribution and Trends of Human Infections

The earliest publication documenting 60 cases of leprosy found that 82.8% originated from the tropical coast, with Guayas province alone accounting for 58.2% of cases. Only five cases were reported from the Andes, and none from the Amazon or Galápagos Islands [15]. A subsequent report describing 120 cases confirmed a similar geographic pattern [17,27].

By 1962, a total of 250 individuals were under treatment across national leprosy care facilities: 120 at the “Verde Cruz” leprosarium in Quito, 45 in “Mariano Estrella” in Cuenca, 30 in the Guayaquil leprosarium, 40 at the Guayaquil Dispensary, and 15 in El Oro province [16]. During a joint active case-finding campaign conducted between 1963 and 1965 by the MoH, PAHO, and UNICEF, 780 new cases were diagnosed, primarily in the coastal provinces of Guayas and Los Ríos, followed by Manabí, Esmeraldas, and El Oro. In the Andes, cases were identified in Azuay, Cañar, Bolívar, Loja, Imbabura, and Pichincha. When including previously reported cases, the national total reached 1120 by June 1965, yielding a prevalence of 0.23 per 1000 people (or 2.26 per 1000 surveyed populations). El Oro province, considered the country’s oldest endemic hotspot, had the highest prevalence (0.98 per 1000), followed by Loja and Guayas [16].

Following the introduction of MDT, a national retrospective study from 1983 to 1990 documented a substantial decline in prevalence—from 2399 cases (0.27 per 1000) in 1983 to 839 cases (0.08 per 1000) in 1990. Incidence rates fell from 1.7 per 1000 in 1983 to 1.01 per 1000 in 1990. By 1990, 94% of cases were concentrated in eight southwestern provinces, which together comprised 66% of Ecuador’s population. High-burden areas were predominantly rural, warm, humid, and mountainous [19].

Between 2014 and 2018, 171 cases were recorded in MoH’s SIVE-ALERTA surveillance system. The majority occurred in Guayas (n = 54; 31.6%), followed by Los Ríos (n = 42; 24.6%) and Loja (n = 23; 13.5%). Thirteen provinces reported cases across Ecuador’s three continental regions, though transmission remained concentrated along the coast. No cases were reported from the Galápagos Islands [28].

A national spatiotemporal analysis using MoH data identified 1539 incident cases reported between 2000 and 2023, showing a progressive decline from 106 cases in 2000 to 42 in 2023. The most affected provinces were Guayas (44.8%), Los Ríos (15.7%), Pichincha (13.1%), El Oro (4.7%), and Loja (4.4%). Eight cantons remained endemic by 2023, with the highest cumulative case counts in Guayaquil (n = 364), Quito (n = 195), Babahoyo (n = 109), Daule (n = 63), Milagro (n = 54), and Loja (n = 45)—with Quito being the only high-burden canton located outside the coast [4,6].

Among the eight published case reports, five originated from coastal provinces, including three from Guayas [7,8,9,10,11,12,23,29]. According to the Global Leprosy Update 2023, Ecuador, with a population of 18.19 million, reported 44 new cases, including 4 non-autochthonous; data on relapses and retreatments were not reported [30].

**Figure 4:**
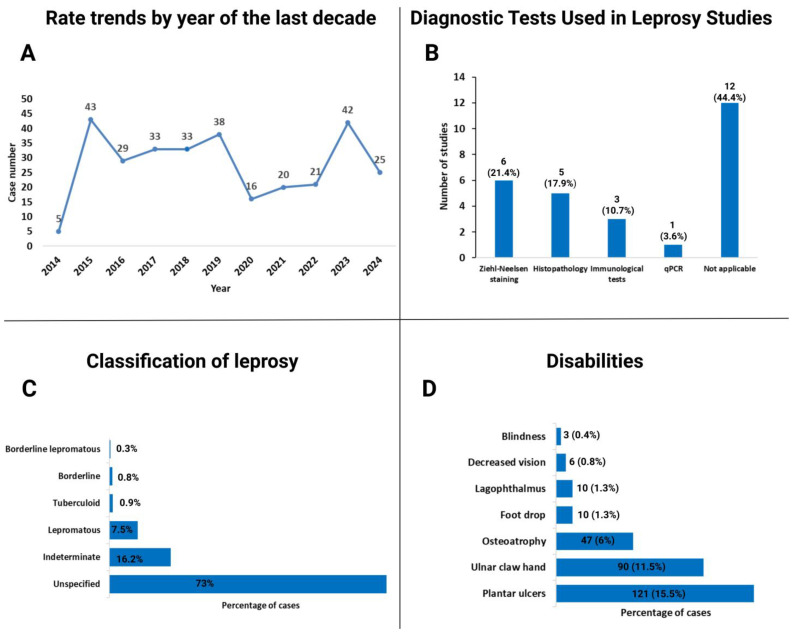
Epidemiological and clinical characteristics of leprosy in Ecuador. (**A**) Annual trends in leprosy notification rates over the past decade (2014–2024), numbers reported by the MoH’s SIVE-ALERTA system. (**B**) Distribution of diagnostic methods employed in leprosy-related studies conducted between 1954 and 2024; immunological tests include the lepromin skin test, anti-PGL-1 and LAM-B antibodies. (**C**) Clinical classification of leprosy reported nationally from 2000 to 2023, according to ICD-10 definitions. (**D**) Proportion of leprosy patients presenting with disabilities during the 1963–1965 national survey.

Finally, based on weekly SIVE-ALERTA bulletins, 124 cases were recorded between 2020 and 2024. The highest annual case count occurred in 2023 (n = 42), followed by 2024 (n = 25). In 2024, twenty cases were diagnosed from the coast (10 in Guayas), four from the Andean provinces (Azuay and Loja), and one from the Amazon [6]. Annual trends for the past decade are presented in Figure 4A.

### 5.3. Demographic Distribution of Leprosy Cases

In the first report of 60 cases, no information was provided regarding sex or age [15]. In the subsequent publication documenting 120 cases, 87 were male and 33 women; regarding age, 7 cases involved children under 15 years of age [17]. In the two-year study (1963–1965), 780 new cases were identified, of which 501 were male (64.2%). A total of 71 cases (9.1%) were reported in individuals aged 0–14 years, including 37 males (4.7%) and 34 women (4.4%). The remaining 709 cases (90.9%) were in individuals over 15 years of age [16]. Between 1983 and 1990, of the 2399 patients, 38.9% were women, and 7.4% were children. Most cases occurred in adults aged 15 years and older, with no cases reported in children under 10. The rate per 100,000 inhabitants among children under 15 years was 1.04 in 1985 and dropped to 0.02 in 1990 [19].

According to data from the MoH’s SIVE-ALERTA (2014–2018), of the 143 cases, 102 were male, resulting in a male-to-female ratio of approximately 2.5:1. The most affected age group was 20–49 years (n = 66; 38.6%), followed by individuals aged ≥ 65 years (n = 51; 29.8%) and those aged 50–64 years (n = 50; 29.2%). One case was reported in a child aged 1–4 years in 2014, and two additional cases were reported in adolescents aged 15–19 years in 2015 and 2016, respectively [28]. In a subsequent report covering 2020–2024, 124 cases were documented, with the highest incidence observed in the 50–64-year age group. Of the 25 cases reported in 2024, 52% were male, and no infections were reported in children [6].

A national analysis using official MoH’s data from 2000 to 2023 documented 1539 incident cases. The median age of affected individuals was 54 years, and 71.5% of cases occurred in males. Notably, 63% of all cases were in individuals aged over 50 years, while children aged ≤ 15 years accounted for only 1.5% of cases. The annual proportion of male cases ranged from 67% in 2000 to 81% in 2022. Over the 23-year period, 23% of cases (n = 612) were in individuals aged ≥ 70 years, and only 1.9% (n = 30) were in children ≤ 15 years old [4]. According to the Global Leprosy Update, 2023, of the 44 new cases reported in Ecuador, 30 were male, and no cases were reported in children [30].

### 5.4. Diagnosis, Laboratory Tests, and Differential Diagnosis

Leprosy diagnosis in Ecuador follows MoH operational case definitions—suspected, clinically confirmed, and laboratory confirmed—in accordance with PAHO guidelines [21,24]. Figure 4B summarizes the diagnostic methods reported in the 28 publications. A serological study in 365 individuals found no correlation between antibody positivity (anti-PGL-I IgM, anti-LAM-B IgG) and disease prevalence, although 3 of 4 confirmed cases were positive for both antibodies [22,23]. Several leprosy cases were initially misdiagnosed as cutaneous and diffuse cutaneous leishmaniasis, and others required differentiation from conditions such as syphilis and superficial mycoses [10,11,15,17,31].

### 5.5. Clinical Features and Classification of Leprosy in Ecuador

Early clinical classification distinguished between malignant (lepromatous) and benign (tuberculoid) forms. In the initial study of 60 cases, 60% were malignant, 36.7% benign, and 3.3% indeterminate [15]. In a follow-up study of 120 cases, 80 were reported as malignant, 36 as benign, and 4 as indeterminate [17]. These studies did not provide details on lesion morphology or anatomical distribution. The average duration of symptoms prior to diagnosis was 8 years, with a range of 1 to 27. During the nationwide active case-finding campaign conducted by the MoH in collaboration with PAHO and UNICEF (1963–1965), 780 new cases were classified using both the Madrid and Ridley–Jopling systems: lepromatous (n = 308; 39.5%), indeterminate (n = 295; 37.8%), tuberculoid (n = 165; 21.2%), and borderline (n = 12; 1.5%). Among the 71 pediatric cases (<15 years), 5% were classified as indeterminate [16].

Following the introduction of MDT, a national study covering the period 1983–1990 reported that 48.2% of newly diagnosed cases were lepromatous [19]. Recent data from the MoH’s SIVE-ALERTA system show that between 2014 and 2018, of the 171 cases, 135 (78.95%) were multibacillary (MB) and 36 (21.05%) paucibacillary (PB); among males, 123 (71.9%) were MB cases [28]. The distribution of clinical forms based on ICD-10 classifications from the national dataset between 2000 and 2023 [4] is shown in Figure 4C. According to the Global Leprosy Update 2023, 39 of the 44 newly reported cases in Ecuador were MB [30]. Among published case reports, one described an atypical ocular presentation involving uveitic glaucoma [9], while three cases documented the rare histoid variant of leprosy [8,10]. Of the 27 cases reported in case reports and series, 55.5% were MB [11,29,32,33].

### 5.6. Etiology, Animal, and Environmental Studies

An investigation identified *M. leprae*, but not *M. lepromatosis*, infecting wild nine-banded armadillos (*Dasypus novemcinctus*). In this study, 18.75% (9/40) armadillos tested positive distributed across all three continental ecoregions of Ecuador. Additional armadillo species sampled, including six *Dasypus* spp. not identified to species level, one *D. pastasae*, and one *Cabassous centralis*, were all negative [25]. A prior study from the coast examined liver samples from a single nine-banded armadillo, and found no acid-fast bacilli [23].

### 5.7. Treatment and Chemoprophylaxis of Leprosy

Chemotherapy in Ecuador, prior to the WHO mandate on MDT, was performed using 100 mg dapsone tablets. The WHO implemented MDT in 1981, but in Ecuador, it was not started until 1983, and by 1987, 100% of patients were compliant with triple therapy [19]. According to the guidelines of the National Health Dermatology Program (1988), the treatments implemented for MB leprosy were dapsone, rifampicin, and clofazimine for 3 years or until bacilloscopies were negative; PB was treated with dapsone and rifampicin for one year [19]. There are no communications on the use of the other chemotherapeutic agents including ofloxacin, minocycline, levofloxacin, sparfloxacin, moxifloxacin, and clarithromycin [34]. On the other hand, we could not find any study/publication related to relapse and/or resistance to any of the drugs used. According to the Global Leprosy Update, 2023, Ecuador, no retreatment or relapse cases were reported. The treatment completion rate was 97% for MB cases and 100% for PB cases [30]. No information on cases of diagnosed type-1 or type-2 reactions was found [21,28].

### 5.8. Control Strategies, Bacillus Calmette–Guérin (BCG) Vaccination

Since the implementation of Ecuador’s national leprosy control in 1967, the MoH has recommended early diagnosis and treatment of all leprosy cases, with a strong emphasis on outpatient and home-based care. Hospitalization was considered voluntary and reserved for selected cases requiring specialized management [16]. The MoH published the Epidemiological Surveillance Subsystem Procedures Manual Alert Action (SIVE-ALERTA), outlining specific prevention and control measures for leprosy [21,28]. The BCG vaccine is included in the national immunization schedule. BCG vaccination is mandatory for all newborns in the country [21,35]. National reports indicate that BCG coverage averaged 86.5% between 2010 and 2020, though declined to 75.3% in 2021 [35].

### 5.9. Disability Outcomes and Social Stigma of Leprosy

The only prospective assessment of disabilities in Ecuador was conducted during the national survey from 1963 to 1965, which evaluated 780 cases using the WHO disability grading system. Of these, 367 individuals (47.1%) were found to have physical disabilities associated with leprosy [16] (Figure 4D). In a more recent retrospective study of 32 patients from El Oro province, 15.6% presented with deformities and 9.4% with disabilities [36]. Two comparative diagnostic studies assessed methods for detecting leprous neuropathy, demonstrating that the pressure-specified sensory device (PSSD) had greater sensitivity than the Semmes–Weinstein monofilament (SWM) and ballpoint pen testing (BPT) [37,38]. Furthermore, a cohort of 19 patients with confirmed leprous neuropathy underwent peripheral nerve decompression surgery as part of a treatment approach to mitigate disability progression [39,40,41]. Notably, no studies have explored the social stigma associated with leprosy in Ecuador.

## 6. Discussion

This review provides the most comprehensive synthesis of the published literature on leprosy in Ecuador from 1954 to 2024, offering updated insights into its epidemiology, research gaps, and strategic priorities. Despite spanning 70 years, only 28 publications were identified, underscoring a persistent neglect of this disease in the national research agenda. Remarkably, no studies have addressed transmission routes, genotyping of *Mycobacterium* species, environmental reservoirs, host genetic susceptibility, antimicrobial resistance, vaccine development, or knowledge, attitudes, and practices (KAP), nor have any explored stigma in affected populations. In alignment with the WHO Global Leprosy Strategy 2021–2030 and the “Towards Zero Leprosy” framework [2,3], Ecuador should tailor interventions to its unique epidemiological context, taking into account regional, ethnic, and linguistic diversity.

Most publications are retrospective, particularly reports from the 1950s and 1960s, which were based on passive case detection at local reference centers [15,17,18,33]. Only one national-level prospective survey using active case-detection was conducted [16]. More recently, an original molecular study detected *M. leprae* DNA in armadillos, suggesting a potential sylvatic reservoir [25]. These findings reveal historically limited research and minimal prioritization of Hansen’s disease in Ecuador, though a modest increase in publications has occurred in recent decades, primarily driven by private sector initiatives.

The increasing number of newly diagnosed cases in Ecuador, from 5 cases in 2004 to 42 in 2023 and 25 in 2024, is a matter of concern. This rise might reflect disruptions caused by the COVID-19 pandemic, including temporary closures of health services and delays in diagnosis. Nevertheless, annual incidence has decreased from 8.5 per million in 2000 to 0.68 per million in 2023 [4]. Globally, in 2023, there was an increase in new case detection in 13 of the 23 global priority countries [5]. In Ecuador, no pediatric cases have been reported since 2014, raising the possibility of interrupted transmission [6,28]. However, this indicator should be interpreted with caution, as newly diagnosed adult cases continue to occur, and zoonotic transmission from infected armadillos, with an estimated 18.75% *M. leprae* infection rate, remains a potential source. Recent adult cases may reflect either childhood infections or delayed diagnosis due to long incubation periods of *M. leprae*.

Ecuador achieved the elimination of leprosy as a public health problem in 2000. However, recent reports from the MoH [6], case reports [7,8,9,10,11,12,23], and findings from a national ecological study [4] confirm that new cases continue to be diagnosed at subnational level, particularly in rural cantons. This pattern suggests that leprosy remains endemic in specific tropical and subtropical areas with limited healthcare access. This scenario aligns with WHO observations that leprosy disproportionately affects individuals living in underserved and remote regions [1]. These findings underscore the need to update Ecuador’s epidemiology.

Historically, most cases have occurred in the tropical coast, particularly in Guayas and Los Ríos provinces, which together account for over half of the 1539 incident cases reported between 2000 and 2023 [4,32,33]. Guayas remains the most endemic province, likely due to its high population density and tropical climate [42]. Notably, no cases have been reported in the Galápagos Islands [4], despite the fact that the population are migrants from endemic regions. This absence highlights the need for active surveillance in previously unreported areas.

Leprosy remains more prevalent among men in Ecuador, possibly due to gender-based environmental exposures such as contact with armadillos during hunting, a known zoonotic reservoir of *M. leprae* [25,43]. This hypothesis requires further investigation and validation. Although leprosy can affect individuals of any sex, this pattern mirrors global trends, where men represent approximately two-thirds of new cases. Globally, the proportion of new cases among men has remained >60% for the past 10 years [5].

To date, despite leprosy’s endemicity in Ecuador, no studies have assessed KAP [4]. Targeted cross-sectional studies using mixed methods are needed to evaluate knowledge gaps in epidemiology, diagnosis, treatment, and prevention. Such research will inform tailored interventions aimed at improving early detection and interrupting transmission. Additionally, we found no studies addressing stigma and discrimination. Exclusion and prejudice hinder access to timely diagnosis, adherence to treatment, and overall healthcare outcomes [2]. Given these implications, it is imperative to conduct prospective studies among patients, healthcare professionals, and in general population. Eliminating discrimination and prejudice is a crucial component of leprosy control and elimination efforts [24]. Education is necessary to elevate the level of awareness in the opinion of community leaders, in the general community, and among traditional healers to eliminate stigma [44]. The occurrence of leprosy in families has led to the misinterpretation that the disease is hereditary [22]. The accumulation of misnomers and misunderstandings could trigger unreasonable reactions in people. Affected people live decades with disability, stigma, and social withdrawal. Disability-adjusted life years (DALY) lost due to leprosy have not been evaluated in the country.

In Ecuador, leprosy diagnosis is concentrated in provincial and specialized hospitals staffed by trained dermatologists [4]. In contrast, rural health centers and cantonal hospitals often lack diagnostic resources and trained personnel [45]. This disparity in healthcare access may contribute to delays in diagnosis and further transmission. It is crucial to enhance the diagnostic capabilities of primary health centers and cantonal hospitals through the provision of appropriate equipment and specialized training for lab technicians and to increase awareness for case detection to community health workers (Promotores de Salud y Técnicos de Atención Primaria en Salud (TAPS) in Ecuador), nurses, and physicians. Furthermore, strengthening referral systems between provincial, cantonal, and rural healthcare facilities is essential to ensure timely diagnosis, treatment, and patients’ adherence. A standardized care package—including BCG vaccination, chemoprophylaxis, education, and physical rehabilitation—should be embedded within primary healthcare services to ensure equity [5].

Most patients likely acquired the infection years earlier in rural areas, given the long incubation period of *M. leprae* (ranging from 2 to 20 years); thus, we recommend the systematic recording of patients’ place of origin or long-term residence to strengthen epidemiological surveillance and guide targeted control efforts. Rural-to-urban migration has resulted in the settlement of displaced populations in urban marginal areas characterized by high population density and overcrowding, conditions that may facilitate transmission in such settings, for example, Guayas canton [42]. Ecuador’s urbanization process accelerated during the 1960s and 1970s, contributing to these demographic shifts in large cities [46].

Another emerging concern in Ecuador is the detection of imported leprosy cases. In 2023, four cases were reported among foreigners [5]. To advance towards leprosy elimination, it is important for health authorities to permit healthcare access for migrants, identifying the nationality and place of origin for future diagnosis purposes. Imported cases should be classified as non-autochthonous as is recommended by WHO [1]. Infected migrants may introduce the bacteria into new areas, particularly if diagnosis is delayed. Symptoms may develop long after migration, posing a sustained risk of transmission within their new communities. Incorporating this epidemiological concept into national reporting systems is critical to accurately track transmission and to refine elimination strategies. Moreover, it would be critical to genotype the *Mycobacterium* strains and testing for antibiotic resistance using molecular–genetic methods [47].

The predominance of MB cases in Ecuador is worrying, as MB leprosy entails higher bacillary load, greater transmission potential, and increased risk of long-term disability. Early publications [17,19], recent surveillance data, and case reports [6,7,10,11,28,33] confirm this trend. Globally, 68.8% of new leprosy cases in 2023 were MB [30]. MB cases pose a considerable challenge to the health system, as extended treatment strain the limited resources of the MoH. The continued detection of advanced cases suggests current surveillance and control measures are inadequate. Active case detection and community-based outreach are essential for timely diagnosis.

Up to 95% of patients exposed to *M. leprae* will not develop the disease, indicating a critical role of host immunity in disease susceptibility and progression. A weakened immune response, often associated with malnutrition, comorbidities, or genetic predisposition, may allow unchecked proliferation of *M. leprae*, resulting in extensive cutaneous and neural involvement typical of MB leprosy. In Ecuador, chronic child malnutrition affects 27.2% of children under two and one in four children under 5 years old [48], likely increasing susceptibility to *M. leprae*. Despite the known role of host genetics in leprosy, no studies in Ecuador have explored genetic predisposition. International evidence points to the involvement of several loci—such as *PARK2*, *NRAMP1*, *TNF*, *TLR1*, *FCN2*, *LTA*, *NOD2*, *RIPK2*, *IL23R*, *RAB32*, *CTSB*, *BATF3*, and *MED30*—in modifying susceptibility to *M. leprae* [34]. Genomic studies, particularly genome-wide association studies (GWAS), should be prioritized in endemic areas. Combining genetic research with epidemiological surveillance and immunological assessments will facilitate the development of targeted interventions and personalized therapeutic strategies.

Leprosy has known animal reservoirs [3,25,49,50], yet no study in Ecuador has investigated transmission sources. This knowledge gap underscores the need for ecological and molecular studies to evaluate zoonotic and environmental transmission pathways, particularly in endemic areas with unexplained human case persistence [33]. Given the presence of at least five armadillo species in the country [51], future studies should include larger, more diverse animal samples and from all over Ecuador. Probable zoonotic transmission of *M. leprae* from armadillos has already been documented in the USA [43]. Hence, research is needed in Ecuador to determine whether *M. leprae* strains found in armadillos can infect humans and whether both share the same genotype. In addition, research studies are deserved to detect *M. leprae* DNA in environmental samples (soil and water) as have been detected in Brazil and India [50]. Viable *M. leprae* in soil have been shown to survive in helminths and amoebas, indicating a potential role of these in bacterial persistence [52]. Researching these interactions from a One Health perspective will provide a more comprehensive understanding of leprosy transmission dynamics in Ecuador.

*M. lepromatosis*, a newly identified species of human leprosy [1] is linked to the severe form of diffuse lepromatous leprosy (Lucio syndrome) [34]. This species has been reported in Mexico, Brazil, Myanmar, and the Caribbean [53]. Further research is warranted in both Ecuadorian patients and potential non-human reservoirs to determine its presence in the country. To date, no studies have been conducted on the genotyping of *M. leprae*. Currently, molecular characterization of *Mycobacterium* species is typically performed using a combination of multiple-locus variable-number tandem repeat analysis (MLVA) and single-nucleotide polymorphism (SNP) typing [54].

In Ecuador, leprosy is misdiagnosed as cutaneous leishmaniasis (CL), syphilis, or superficial fungal infections [10,11,23,31]. CL and mucosal leishmaniasis co-occur in the tropical regions of the coastal and Amazon areas of Ecuador [23,55]. Histopathological examination of biopsied inflamed peripheral nerves, revealing characteristic neural inflammation, can aid in distinguishing leprosy from other granulomatous diseases [56]. However, no official publications have addressed the use of nerve biopsy for diagnosis. Additionally, clinicians in the Andes region rarely consider leprosy, contributing to diagnostic delays. We recommend incorporating leprosy into differential diagnoses for chronic skin, ocular, and peripheral neuropathy symptoms, and providing continuous training to healthcare workers—both public and private—through MoH and academic platforms. Only one case report informed the eye damage [9] that is frequently seen in MB patients resulting from both nerve damage and direct bacillary invasion [34].

Despite being widely implemented in developed countries, molecular diagnosis of leprosy by polymerase chain reaction (PCR) [34], it remains unavailable in Ecuador, both in public and private laboratories. The National Institute of Public Health Research (INSPI), as the country’s reference laboratory, should prioritize the implementation of PCR for Hansen’s disease, particularly since it is already performed for other pathogens such as *M. tuberculosis*, avian Influenza A virus, etc. [57]. Antibodies (anti-PGL-1 and anti-LAM-B) detection were used in Ecuadorian patients and their household contacts [22]. Nevertheless, the relatively low specificity of these antibodies has led to their discontinuation in clinical practice [34]. Testing novel synthetic peptides (e.g., PEP1 to PEP8) could enhance the sensitivity for early detection of the infection [58]. Training laboratory technicians and pathologists in sample collection, Ziehl–Neelsen staining, and microscopic identification of *M. leprae* should be prioritized. These efforts could be integrated into broader capacity-building initiatives for other neglected tropical diseases (NTDs) like leishmaniasis, Chagas disease, dengue, and malaria. Given the widespread availability of Ziehl–Neelsen staining for tuberculosis diagnosis in Ecuadorian health centers [59], its application for leprosy diagnostics could be rapidly scaled up at minimal cost. Yet, despite its simplicity and affordability, leprosy remains a neglected disease, with diagnostic delays averaging 8 years [4,7,19].

No official data or publications are available on leprosy reactions in Ecuador. The management of leprosy reactions, type 1 (reversal reactions) and type 2 (erythema nodosum leprosum), require prompt clinical recognition and targeted pharmacologic treatment, as these represent acute immunological exacerbations that may arise before, during, or after MDT [34]. National official guidelines should provide recommendations for patient care, such as referrals for ophthalmologic, surgical, and psychological evaluations, or for specific physical rehabilitation services. Furthermore, we were unable to identify any official information regarding the rate of leprosy infection among household contacts of index cases. This gap is reflected in Ecuador’s absence from the 88 countries that reported contact tracing activities to the WHO [5].

Drug-resistant *M. leprae* is a global concern [60]. However, there is currently no data on antimicrobial susceptibility of *M. leprae* in Ecuador. Research on antimicrobial resistance should be a priority in Ecuador, since MDT was initiated in 1993. Countries are encouraged to strengthen antimicrobial resistance surveillance for the three drugs used, especially with scaling-up the post-exposure prophylaxis (PEP) using a single dose of rifampicin (SDR) [5]. Drug resistance of *M. leprae* is primarily investigated through molecular techniques and computational analyses [60,61]. In cases of resistance, alternative drugs such as fluoroquinolones and macrolides have been utilized [34]. Additionally, the novel drug bedaquiline has demonstrated promising efficacy as monotherapy in MB cases [62]. Given these developments, we recommend the design and implementation of clinical trials to evaluate the efficacy of these alternative regimens in Ecuadorian patients, including both MB- and PB-resistant cases.

Standard MDT may be associated with adverse drug reactions (ADRs); there are no reports on this topic in Ecuador. We encourage strengthening mechanisms for early identification, effective management, and timely reporting of ADRs following the recommendations of the WHO [5]. Furthermore, data on treatment completion or cases of re-treatment is also not available; this should be published for MB and for PB cases. Better understanding of the reasons for non-adherence to treatment will improve targeted interventions and ensure efficient MDT services. Uncompleted treated cases contribute to developing resistance and prolonged transmission within their households and social networks [2]. In addition, there has been little or no monitoring of clinical outcomes and relapse rates [5]; accurate diagnosis of relapse requires clinical, bacteriological, and histopathological evidence that need to be provided at cantonal hospitals.

Historical data on leprosy-related disabilities in Ecuador is scarce [16]. Recent MoH reports fail to include disability data [21,28,63]. Ecuadorian guidelines should align with WHO disability grading criteria [64]. Prompt diagnosis and management of leprosy reactions and neuritis is effective in preventing or minimizing the development of further disabilities [37,38]. Although physical rehabilitation is recommended [21], access remains inadequate, particularly in rural and underserved areas where financial and geographic barriers impede access. To address these disparities, the MoH should prioritize the integration of rehabilitation services into provincial and even at cantonal hospitals. Comprehensive disability management should include medical and surgical treatments such as ulcer care, reconstructive procedures, e.g., nerve decompression for leprous neuropathy [40,41]. Disability services should encompass trained physical therapists, access to assistive technologies, and reconstructive surgical interventions.

It is advisable for Ecuador to follow the WHO “Leprosy/Hansen’s disease: Contact tracing and post-exposure prophylaxis” publication [65] on the post-exposure prophylaxis (PEP) using a single dose of rifampicin (SDR) to all contacts of confirmed cases. PEP-SDR has shown ~57% efficacy in reducing leprosy incidence among contacts and has contributed to case reductions in several countries [1,2]. Implementation in Ecuador would be feasible using existing public health infrastructure. The MoH should monitor coverage, rifampicin availability, and program outcomes to optimize impact. Strategies to prevent leprosy include BCG vaccination [1]. Ecuador’s national immunization schedule includes BCG for all newborns, but coverage fell to 75.3% in 2021, likely due to COVID-19-related disruptions in health services [35]. The MoH must strengthen efforts to maintain high BCG coverage, not only as a tuberculosis intervention, but also as a strategy to mitigate the severity and transmission of leprosy. Recent reports of non-autochthonous cases further underscore the need for strengthened surveillance on BCG vaccination among migrant populations. The MoH should provide updated and on-time coverage BCG vaccination rates as is solicited by the PAHO/WHO [1].

Leprosy surveillance in Ecuador is conducted through the SIVE-ALERTA system, utilizing its online platform ViEpi. This system records and reports leprosy cases nationwide, collects epidemiological data from provincial health services, and publishes weekly updates. However, surveillance data are not disaggregated to the subnational level, such as canton or parish levels, which would be highly desirable for targeted interventions. The current MoH system lacks critical information, including the probable site of infection, degree of disability at diagnosis, occurrence of leprosy reactions, end-of-treatment assessments, and progression or worsening of disability grades. Strengthening the surveillance system is essential to achieving the goals outlined in the WHO “Towards Zero Leprosy: Global Leprosy (Hansen’s Disease) Strategy 2021–2030.”

## 7. Conclusions and Future Perspectives

Since the introduction of MDT in 1983, leprosy prevalence in Ecuador has steadily declined, reflecting significant progress toward national elimination goals. To consolidate these gains and move toward interruption of transmission, the MoH should implement integrated strategies that combine preventive measures, such as BCG vaccination and single-dose rifampicin chemoprophylaxis for contacts with active case detection, early treatment, capacity building for health personnel, and community-based education. These efforts should be aligned with existing public health initiatives, including national immunization and soil-transmitted helminth control programs, to optimize resource use and coverage. Improved case surveillance incorporating parish-level geospatial data is essential to better target interventions and monitor transmission dynamics. A national strategy that integrates surveillance, diagnosis, treatment, disability prevention, rehabilitation, and public awareness is critical to achieving the milestones set by the WHO Roadmap for Neglected Tropical Diseases 2021–2030. Furthermore, adopting a One Health perspective to explore zoonotic and environmental reservoirs may help address potential sources of persistent transmission. Closing current knowledge gaps through multidisciplinary research and intersectoral collaboration will be vital for Ecuador to achieve and sustain a leprosy-free status. Although national prevalence remains below the WHO elimination threshold, continued political commitment, funding, and coordinated actions across all levels of the health system are necessary to achieve zero leprosy.

## Data Availability

Supplemental Table S1. Publications on leprosy in Ecuador from 1954 to 2024 (70-year period).

## Supplementary Materials

The following supporting information can be downloaded at: https://www.mdpi.com/article/10.3390/pathogens14080832/s1, Table S1: Publications on leprosy in Ecuador from 1954 to 2024 (70-year period). The 28 articles are listed chronologically by year of publication.
