# Supplementary material for: Hansen’s Disease in Ecuador: Current Status, Knowledge Gaps, and Research Priorities: A Literature Review
Source: Pathogens. 2025 Aug 21;14(8):832. doi: 10.3390/pathogens14080832 (PMC12389554; doi:10.3390/pathogens14080832)
Supplement: Supplementary file 1 [file pathogens-14-00832-s001.zip › pathogens-3823120-supplementary.pdf]

Supplemental Table S1. Publications on leprosy in Ecuador from 1954 to 2024 (70-year period). The 28 articles are listed chronologically by year of publication.

**First Author/ Publication Year**	**Article Type**	**Ecuador´s Ecoregion/ Provinces**	**Language/ Journal target Research Design (Temporal Dimension)**	**Case Numbers**	**Leprosy Type (N Cases)**
1. Aviles F, & Blum-Gutiérrez E, 1954	Original Research	Coast & Andes/ Guayas Los Rios El Oro Cañar Azuay Bolivar Manabi Esmeraldas	Spanish/National Retrospective	60	Lepromatous (36) Tuberculoid (22) Indeterminate form (2)
2. Blum-Gutierrez E, 1954	Case series	Coast/ Guayas	Spanish/National Retrospective	16	Lepromatous (8) Tuberculoid (5) Indeterminate form (2) Unknow (1)
3. Aviles F, & Blum-Gutiérrez E, 1956	Original research	Coast & Andes/ Guayas, El Oro, Los Rios, Manabi, Esmeraldas, Azuay, Bolivar, Loja, Cañar	Spanish/National Retrospective	120	Lepromatous (80) Tuberculoid (36) Indeterminate form (4)
4. Blum-Gutierrez E, 1956	Original research	Coast	Spanish/National Retrospective	1341	NA
5. Blum-Gutierrez E, 1957	Review	Nationwide	Spanish/National Retrospective	NA	NA
6. Blum-Gutierrez E, 1966	Original research	Nationwide	Spanish/National Retrospective	978	Lepromatous (381) Tuberculoid (200) Indeterminant (374) Dimorphic (23)
7. Blum-Gutierrez E, 1967	Original Research	Coast & Andes/ Esmeraldas Manabi Guayas Los Rios El Oro Loja Azuay Cañar Bolivar Pichincha Imbabura	Spanish/National Prospective	780	Lepromatous (308) Indeterminate (295) Tuberculoid (165) Dimorphic (12)
8. Zarate N, 1992	Original research	Nationwide	Spanish/National Retrospective	13632	Lepromatous Tuberculoid Dimorphic Indeterminate form (Does not specify the number of cases)
9. Hosokawa A[b], 1994	Original research	Coast & Andes/ Manabí Bolívar El Oro Guayas Esmeraldas	English/International Prospective	154	Lepromatous (3) Tuberculoid (3) Indeterminate form (6) Unknow Type (1)
10. Hosokawa A[a], 1994	Case series	Coast/ Manabi	English/International Prospective	4	Borderline lepromatous (2),

					indeterminate (1), unknow (1)
11. Terán S, 2010	Original research	Coast/ Guayas	Spanish/National Retrospective	25	Lepromatous (14) Dimorphic (5) Indeterminate form (6)
12. Franco J, 2012	Thesis	Coast/ El Oro	Spanish/National Prospective	5	Lepromatous (5)
13. Baltodano PA, 2015	Original research	Coast/ Los Rios	English/International Prospective	71	Lepromatous neuropathy
14. Baltodano PA, 2016	Original Research	Coast/ Los Rios	English/International Prospective	39	Lepromatous neuropathy
15. Dellon AL, 2016	Original Research	Coast/ Guayas	English/International Retrospective	32	NA
16. Wan E, 2016	Original research	Coast/ Guayas	English/International Prospective	12	NA
17. Wan E, 2017	Original Research	Coast/ Guayas	English/International Prospective	19	NA
18. Polo-Checa AM, 2017	Original research	Coast/ El Oro	Spanish/National Prospective	32	Lepromatous (19) Tuberculoid (7) Indeterminate form (6)
19. Dávila-Rodríguez J, 2019	Case series	NA	Spanish/International Retrospective	2	Lepromatous (2)
20. Egas J, 2019	Thesis	NA	Spanish/National NA	NA	NA
21. Moncada D, 2019	Thesis	Nationwide	Spanish/National NA	NA	NA
22. Baquero-Suárez J, 2019	Case report	Coast/ Guayas	Spanish/International Retrospective	1	Lepromatous (1)
23. Roldan-Vasquez A, 2021	Case report	NA	English/International Retrospective	1	Tuberculoid (1)
24. Martínez P, 2021	Case report	Coast/ Guayas	Spanish/National Retrospective	1	Lepromatous (1)
25. Hernandez-Bojorge S, 2024	Original research	Nationwide	English/International Retrospective	1539	Lepromatous (116) Tuberculoid (14) Unspecified (1123) Other (3)
26. Romero-Alvarez D, 2024	Original research	Coast, Andes & Amazon/ Imbabura Esmeraldas Manabí Santo Domingo Cañar Sucumbíos/ Pastaza	English/International Prospective	48	NA

27. Polanco CRA, 2024	Case report	Coast/ Manabi	Spanish/International Retrospective	1	Lepromatous (1)
28. Ramírez-Honores J, 2024	Case report	Amazon/ Unknown	Spanish/International Retrospective	1	Lepromatous (1)

NA: not applicable.
